# Ligand-Induced Variations in Structural and Dynamical Properties Within an Enzyme Superfamily

**DOI:** 10.3389/fmolb.2018.00054

**Published:** 2018-06-12

**Authors:** Chitra Narayanan, David N. Bernard, Khushboo Bafna, Donald Gagné, Pratul K. Agarwal, Nicolas Doucet

**Affiliations:** ^1^INRS – Institut Armand-Frappier, Université du Québec, Laval, QC, Canada; ^2^Genome Science and Technology, University of Tennessee, Knoxville, TN, United States; ^3^Computational Biology Institute and Computer Science and Engineering Division, Oak Ridge National Laboratory, Oak Ridge, TN, United States; ^4^Department of Biochemistry, Cellular and Molecular Biology, University of Tennessee, Knoxville, TN, United States; ^5^PROTEO, The Québec Network for Research on Protein Function, Engineering, and Applications, Université Laval, Québec, QC, Canada

**Keywords:** enzyme catalysis, ligand binding, chemical shift projection analysis, CHESPA, pancreatic ribonucleases, nuclear magnetic resonance, titration

## Abstract

Enzyme catalysis is a complex process involving several steps along the reaction coordinates, including substrate recognition and binding, chemical transformation, and product release. Evidence continues to emerge linking the functional and evolutionary role of conformational exchange processes in optimal catalytic activity. Ligand binding changes the conformational landscape of enzymes, inducing long-range conformational rearrangements. Using functionally distinct members of the pancreatic ribonuclease superfamily as a model system, we characterized the structural and conformational changes associated with the binding of two mononucleotide ligands. By combining NMR chemical shift titration experiments with the chemical shift projection analysis (CHESPA) and relaxation dispersion experiments, we show that biologically distinct members of the RNase superfamily display discrete chemical shift perturbations upon ligand binding that are not conserved even in structurally related members. Amino acid networks exhibiting coordinated chemical shift displacements upon binding of the two ligands are unique to each of the RNases analyzed. Our results reveal the contribution of conformational rearrangements to the observed chemical shift perturbations. These observations provide important insights into the contribution of the different ligand binding specificities and effects of conformational exchange on the observed perturbations associated with ligand binding for functionally diverse members of the pancreatic RNase superfamily.

## Introduction

Enzyme catalysis accelerates reaction rates of chemical reactions up to 20 orders of magnitude relative to uncatalyzed reactions (Wolfenden, 2006). In the widely understood paradigm, enzymes act by reducing the free energy barrier, thus facilitating the formation of the transition state. The mechanism of enzyme catalysis is complex, including, but not limited to, the following steps: substrate recognition and binding to the active site; the chemical step involving the conversion of substrate(s) to product through the transition state; and release of product(s). Any of these steps along the reaction coordinates could act as the rate-limiting step, thus determining the rate of enzyme turnover (Gutteridge and Thornton, 2004; Narayanan et al., 2016). More recently, conformational rearrangements, corresponding to time-dependent atomic displacements of residues and/or larger structural elements, have been suggested to influence the catalytic power of enzymes (Pelz et al., 2016; Kovermann et al., 2017). This conformational flexibility, while maintaining the native three-dimensional structure of the protein, is often essential for optimal enzyme function (Henzler-Wildman et al., 2007). Enzymes were shown to sample distinct conformations, termed *sub-states*, facilitated by conformational fluctuations that occur over a wide range of timescales (Narayanan et al., 2016). While the role of conformational exchange in enzyme catalysis is debated, evidence from experimental and computational approaches have revealed the correlation between enzyme turnover rates and the timescale of conformational motions in numerous enzyme systems, including but not limited to, alcohol dehydrogenase, dihydrofolate reductase, and ribonuclease A (RNase A) (Agarwal et al., 2012; Narayanan et al., 2016).

**Figure 1:**
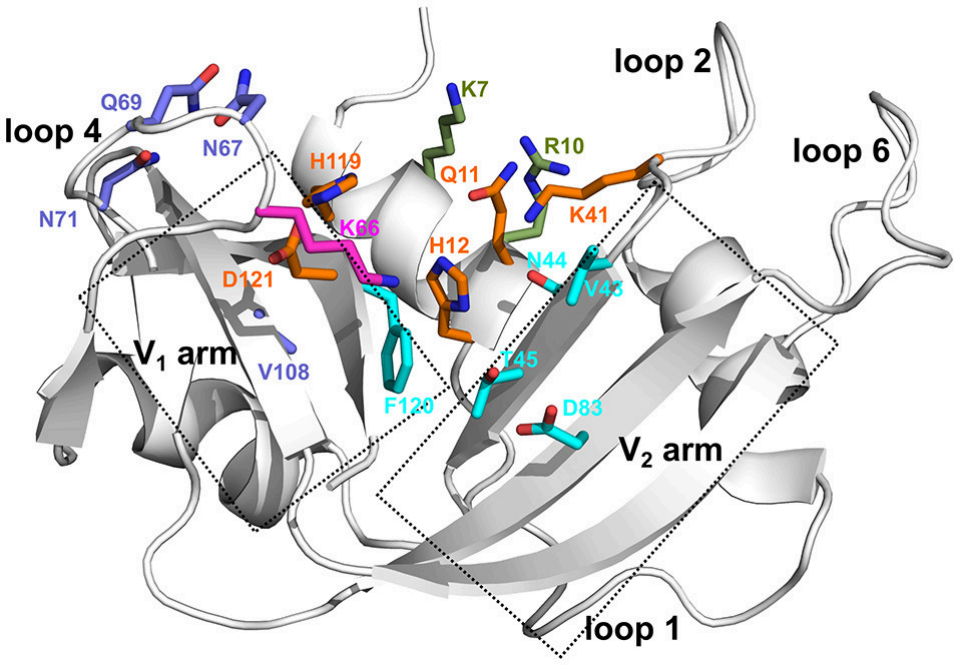
Ligand binding subsites identified in bovine RNase A. Residues of the ligand binding subsites, corresponding to the base B_1_, B_2_, and phosphate P_0_, P_1_, and P_2_ subsites are represented as sticks colored cyan, purple, magenta, orange, and olive, respectively. Catalytic residues His12, Lys41, and His119 form part of the P_1_ binding site.

Members of the pancreatic ribonuclease superfamily have served as a model system for numerous biophysical experiments, including enzyme mechanism studies (Sorrentino, 2010). Bovine RNase A is the prototypical member of this superfamily, whose primary function is the cleavage of the 3′,5′-phosphodiester bond in single- and double-stranded RNA substrates. Several phosphate (P_n_) and nucleotide base (B_n_) binding subsites that interact with the substrate molecules were identified in the active site of bovine RNase A (Figure 1), which displays a strong preference for a pyrimidine in the primary base binding site (B_1_) and a purine in the secondary base binding site (B_2_) (Nogués et al., 1995). The rate-limiting step was previously shown to correspond to a conformational change in a distal loop that is associated with the product release step in RNase A (Watt et al., 2011; Gagné and Doucet, 2013). The functional role of conformational exchange in product release was previously shown to rely on the movement of distal loop regions in RNase A, a hypothesis that we further extended to include functional RNase homologs sharing a conserved structural fold (Cole and Loria, 2002; Watt et al., 2007; Doucet et al., 2009, 2011; Gagné et al., 2012; Gagné and Doucet, 2013; Narayanan et al., 2017, 2018). Mutations of residues in these loop regions were shown to result in reduced rate constants for product release and lower substrate affinity, highlighting the role of these long-range motions in this enzyme (Gagné and Doucet, 2013). Eight catalytically active (canonical) and five inactive (non-canonical) RNases were identified in the sequencing of the human genome (Cho et al., 2005). In addition to their common ribonucleolytic function, the canonical RNases, henceforth referred to as *subtypes*, have evolved to perform other biological functions such as host defense, immunosuppressivity, angiogenesis, and anti-pathogenic activity, among others (Sorrentino, 2010). Further, the experimentally characterized human RNase subtypes display a wide range of substrate specificities (Boix et al., 2013), catalytic activities (Sorrentino, 2010; Gagné and Doucet, 2013) and conformational fluctuations on the millisecond timescale (Narayanan et al., 2017, 2018). Efforts to relate specific conformational exchange events with ribonucleolytic function in this enzyme family is thus limited by the broader and often RNA-independent biological functions of many homologous RNase superfamily members.

Coevolving amino acid residues that are proximal to each other in the three-dimensional structure may control the biological properties of proteins (Halabi et al., 2009). In a recent study, we identified networks of coevolving amino acids that control distinct aspects of biochemical functions in the pancreatic RNase superfamily by combining the sequence-based statistical analysis with experimental observations (Narayanan et al., 2017). Our results demonstrated that networks of residues within a larger functional sector are involved in fine-tuning the catalytic activity among the different RNase subtypes, thus dictating the functional diversity among these RNases. Detailed characterization of the dynamical properties of over 20 RNases in the ligand-free states further revealed large differences in their global conformational exchange patterns observed for members within the superfamily (Narayanan et al., 2018). Using a diverse set of RNases grouped into functionally distinct phylogenetic subfamilies, we demonstrated the conservation of conformational exchange profiles between RNases within subfamilies sharing similar biological functions, while significant differences were reported between subfamilies (Narayanan et al., 2018). These observations from characterization of the ligand-free (*apo*) state of enzymes provided important insights into the selective pressure that may influence the exchange profiles within a superfamily.

Ligand binding influences the conformational landscape of enzymes, leading to structural and dynamical changes. However, these changes remain largely uncharacterized for most RNases. We previously showed the long-range effects of ligand binding on the structural and dynamical behaviors of select RNases (Gagné et al., 2012, 2015b). While these studies provided important insights into the effect of ligand binding to these RNases, several important questions remain unanswered, which the present study seeks to address. For instance, how do structural and conformational exchange properties of different enzymes within the superfamily change upon ligand binding? Do members within functionally distinct subfamilies display similar conformational rearrangements upon ligand binding, as observed for the ligand-free states of members of the RNase superfamily? This study aims to compare and characterize the structural and conformational changes associated with ligand binding for select RNases, corresponding to representative family members with distinct biological functions, and to gain insights into the mechanism of ligand binding for members of the pancreatic RNase superfamily.

In this study, we probe the chemical shift changes associated with the binding of two mononucleotide ligands (3′-UMP and 5′-AMP) to five selected RNases, corresponding to representative members of four distinct phylogenetic subfamilies. The two mononucleotides mimic the hydrolysis products of the model RNA dinucleotide substrate UpA. Our results show that binding of each of the two ligands induces distinctly different effects on selected RNases. Despite their local and global structural similarity, RNases from functionally distinct subfamilies displayed different effects upon binding to each of the two ligands. Further, RNases within a subfamily also displayed different magnitudes of chemical shift changes near the active site, in addition to notable differences in the long-range effects of the ligand binding subsite. We determined the coordinated changes in chemical shift displacements associated with the binding of the two ligands for the selected RNases using the NMR chemical shift projection analysis (CHESPA). Our results show that networks of residues displaying coordinated displacements vary in RNases within and between different subfamilies. Our results further illustrate the contribution of dynamical changes upon ligand binding to the observed chemical shift perturbations. We suggest that, among other factors, the distinct nucleotide binding specificities and conformational rearrangements triggered by ligand binding may be contributing to the unique functional and biological roles of these RNases within the cell, despite their apparent structural similarity.

## Methods

### Enzyme cloning, expression and purification

Bovine RNase A and human RNases 2, 3, 4, and 5 were cloned, expressed, and purified according to protocols described previously (Doucet et al., 2009; Gagné et al., 2012, 2015b). Sequences were codon-optimized for *Escherichia coli* expression and cloned into *Nde*I/*Hin*dIII-digested expression vector pJexpress411 (ATUM, Newark CA). ^15^N- and [^15^N/^13^C]-labeled protein expression and purification was performed using previously described protocols (Doucet et al., 2009; Gagné et al., 2012), with these modifications: the temperature was lowered to 30°C following addition of IPTG, the volume of culture media was 1 L, and bacteria were grown overnight before being harvested by centrifugation. Protein concentrations were determined using extinction coefficients of 9,880 (RNase A), 17,460 (RNases 2, 3), and 11,835 (RNases 4, 5) M^−1^cm^−1^, respectively, as estimated by ExPASy ProtParam.

### Solution NMR experiments

2D ^1^H-^15^N HSQC, 3D-HNCACB and 3D-CBCA(CO)NH assignment experiments were performed using a Varian INOVA 500 MHz (11.7 T) spectrometer at 298 K. NMR data processing and analyses were performed using NMRPipe (Delaglio et al., 1995), CcpNmr Analysis (Vranken et al., 2005), and Sparky (Goddard and Keneller, 2008).

### NMR titration experiments

All NMR titration experiments were conducted at 298 K on ^15^N-labeled 150–450 μM protein samples in 15 mM sodium acetate at pH 5.0. The pH was carefully monitored throughout the experiments and readjusted with Tris-base or acetic acid if necessary. Ligands 3′-UMP (Chemical Impex Intl Inc., Wood Dale, IL, USA) and 5′-AMP (BioBasic Inc., Markham, ON Canada) were purchased commercially and dissolved in the same buffer as the protein. ^1^H-^15^N sensitivity-enhanced HSQC experiments were acquired at 800 MHz (18.8 T) using spectral widths (points) of 2025 (160) and 8000 Hz (1024) in the ω_1_ and ω_2_ dimensions, respectively. Titration experiments were performed for each ligand with enzyme:ligand molar ratios up to 1:12 (1:12), 1:18 (1:18), 1:24 (1:18), 1:30 (1:18) and 1:18 (1:30) for the 3′-UMP- (5′-AMP-) bound states of bovine RNase A and human RNases 2, 3, 4, and 5, respectively.

The equilibrium dissociation constant (*K*_*d*_) was calculated by plotting weighted average chemical shift differences (Δδ_obs_) as a function of ligand concentration and fitting the data using the following equation:

(1) $$\begin{array}{l} \Delta \delta_{obs}=\frac{\Delta \delta_{\max}}{2{\left[P\right]}_{t}}({\left[P\right]}_{t}+\frac{{\left[L\right]}_{t}}{{\left[P\right]}_{t}}{\left[P\right]}_{t}+K_{d}\\ \;-\sqrt{{\left({\left[P\right]}_{t}+\frac{{\left[L\right]}_{t}}{{\left[P\right]}_{t}}{\left[P\right]}_{t}+K_{d}\right)}^{2}-4\frac{{\left[L\right]}_{t}}{{\left[P\right]}_{t}}{{\left[P\right]}^{2}}_{t}}) \end{array}$$

The *K*_*d*_ was estimated by simultaneously fitting the data of all residues affected by ligand binding (Williamson, 2013). We note that Δδ_max_ is a fitted parameter and not an experimentally measured one. Uncertainties on these values are given by the standard deviations of the fits. Chemical shift perturbations (Δδ_obs_) were calculated as the difference in the weighted average chemical shift of the ligand-bound (5′-AMP and 3′-UMP) and apo states, as shown below.

(2) $$\begin{array}{l} \Delta \delta_{obs}=\sqrt{\frac{{\left(\Delta \delta_{H}\right)}^{2}+{\left(0.2\Delta \delta_{N}\right)}^{2}}{2}} \end{array}$$

### ^15^N-carr-purcell-meiboom-gill (CPMG) NMR relaxation experiments

Relaxation dispersion experiments for the apo and 3′-UMP- or 5′-AMP-saturated enzyme complexes were performed using published methods (Doucet et al., 2009) and pulse sequences (Loria et al., 1999). Experiments were carried out on 500 MHz (11.7 T) and 800 MHz (18.8 T) Varian (Agilent) NMR spectrometers equipped with a triple-resonance cold probe and pulsed-field gradients. Interleaved two-dimensional spectra were collected in a constant time manner with τ_cp_ repetition delays of 0.625, 0.714 (×2), 1.0, 1.25, 1.67, 2.0, 2.50 (×2), 3.33, 5.0, and 10 ms, within a total relaxation period of 40 ms. NMR spectra were processed using NMRPipe (Delaglio et al., 1995), analyzed with Sparky (Goddard and Keneller, 2008) and in-house CPMG scripts. Collected data was dual-fitted to the Carver-Richards full relaxation dispersion equation (Manley and Loria, 2012).

### Definitions for (un)coordinated dynamical changes

Residues which showed a difference between measured *R*_2_ (1/τ_cp_) values at fast (τ_cp_ = 0.625 ms) and slow (τ_cp_ = 10 ms) refocusing pulse delays greater than 2 s^−1^ were considered for further analysis, similar to previous studies (Gagné et al., 2012, 2015b). We compared these residues which show relaxation dispersion curves with Δ*R*_2_ > 2 s^−1^ for the 3′-UMP- and 5′-AMP-bound states with that of the apo state to identify residues displaying (un)coordinated dynamical changes. Residues that show a gain (or loss) of millisecond exchanges in *both* the 3′-UMP- and 5′-AMP-bound states relative to the apo state are defined as displaying *coordinated* changes in motions. Residues displaying a gain (or loss) of dynamics in *only one* of the two (5′-AMP or 3′-UMP) ligand-bound states relative to the apo state are defined as displaying *uncoordinated* changes. We perform a qualitative comparison of the residues displaying (un)coordinated changes in dynamics by comparing residues that show loss (or gain) of conformational exchange in either or both ligand-bound states relative to the apo state of the enzymes, respectively. Consequently, the current interpretation reports on qualitative changes in relaxation dispersion profiles between enzyme states and does not presume to quantitatively describe changes in the sign and direction of chemical shifts between two similar relaxation dispersion profiles.

### Chemical shift projection analysis (CHESPA)

CHESPA was performed based on the protocol described by Selvaratnam et al. (2012). The chemical shift perturbations (Δδ_obs_) of the ligand-bound state relative to the apo state correspond to the shifts for the highest enzyme:ligand molar ratios for each enzyme. For each of the five RNases, residues with a chemical shift variation Δδ_obs_ > 0.05 ppm were selected for further analysis. The two CHESPA parameters, projection angle (cos(θ)) and fractional shift (X), were calculated according to the equation below using the ^1^H and ^15^N peak coordinates in their free (apo) form and upon binding to the two ligands, 3′-UMP (vector A) and 5′-AMP (vector B) at saturation conditions.

(3) $$\begin{array}{l} \cos\left(\theta \right)=\frac{\text{A}\cdot \text{B}}{\left|\text{A}|\cdot |\text{B}\right|},X=\frac{\text{A}\cdot \text{B}}{{\left|\text{B}\right|}^{2}} \end{array}$$

### Bioinformatics analyses

Multiple sequence alignment of the bovine and Hominidae RNases 1–8 sequences in fasta format was performed using ClustalΩ (Sievers et al., 2011). Phylogenetic analysis of all RNases was performed using the maximum likelihood approach with RaxML v8.0.26 and the WAG amino acid substitution model (Stamatakis, 2014). Bootstrapping analysis over 100 iterations was used to assess the reliability of the branching. Figtree, v1.4.2 (http://tree.bio.ed.ac.uk/software/figtree/) was used to visualize the phylogenetic tree input in newick format.

## Results

### Evolutionary relationship between sequences

**Figure 2:**
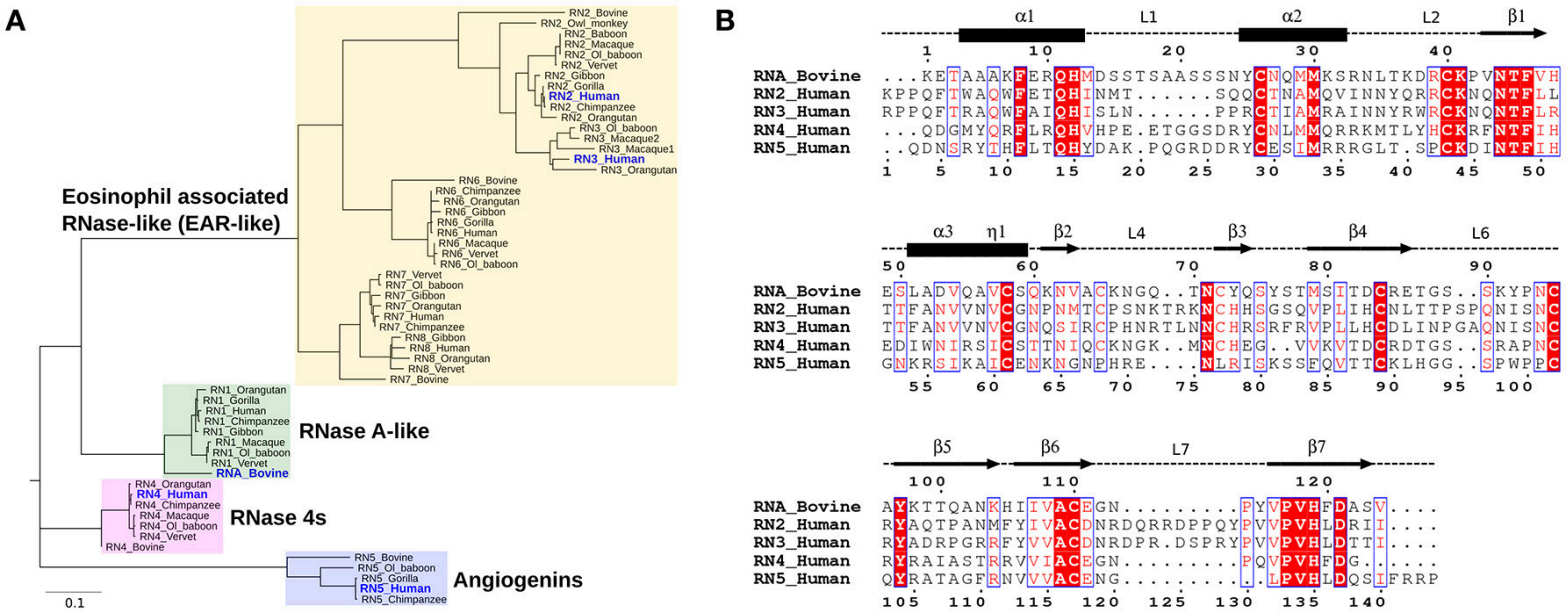
Sequence characterization of pancreatic-type RNases. **(A)** Phylogenetic clustering of Hominidae and bovine RNases 1–8 groups RNases into four *subfamilies*. The multiple sequence alignment of all RNases used for this analysis is shown in Figure S2. **(B)** Multiple sequence alignment of bovine RNase A (RNA_Bovine), human RNases 2 (RN2_Human), 3 (RN3_Human), 4 (RN4_Human), and 5 (RN5_Human). The primary sequence numbering for RNase A and the consensus sequence, corresponding to sequence numbering including gaps in the alignment, are shown in the top and bottom of the alignment, respectively. Secondary structure of bovine RNase A is traced on top of the sequence alignment using blocks, arrows and dashed lines to represent α-helix, β-strand and loop regions, respectively. Sequence alignments were prepared using EsPript (Robert and Gouet, 2014).

Phylogenetic analyses provide important insights into the evolutionary determinants of the structural and functional diversity within an enzyme family. Here, we performed the phylogenetic classification of the eight canonical RNase subtypes from bovine and Hominidae members (Figure 2). Phylogenetic clustering led to grouping of these sequences into distinct *subfamilies*, whereby sequences within subfamilies share similar biological functions, consistent with previous observations (Narayanan et al., 2018). The eosinophil-associated RNase-like (EAR-like) homologs in human were shown to display antiviral and antimicrobial activities, while angiogenins were named after their role in angiogenesis (Koczera et al., 2016). Human RNase 4 was shown to be expressed in host-defense associated tissues, while the primary function of RNase A-like sequences involves the degradation of RNA (Koczera et al., 2016). RNases selected for this study share an average pairwise sequence identity of 37% and are identified in blue in Figure 2A.

We selected five representative members from the four functionally distinct phylogenetic subfamilies to characterize the effect of ligand binding on the structural and dynamical properties of these functionally distinct RNases. The selected enzymes are bovine RNase A (RNA_Bovine), human RNase 2 (RN2_Human), RNase 3 (RN3_Human), RNase 4 (RN4_Human) and RNase 5 (RN5_Human). Multiple sequence alignment of the selected sequences showed the conservation of residues in the active site (His12, Lys41 and His119, RNase A numbering) and other residues associated with substrate binding and discrimination in RNase A, including Thr45 (B_1_ pyrimidine binding subsite), Asn71 (B_2_ purine binding subsite) and phosphate binding subsites Gln11, Asp121 (Figure 2B). Other ligand binding subsites identified in RNase A, such as Lys7, Arg10, Lys66, and Asp83 (Raines, 1998), are not conserved across other RNases, suggesting a potential effect on the substrate specificity and affinity in these RNases.

### Chemical shift changes associated with ligand binding

**Figure 3:**
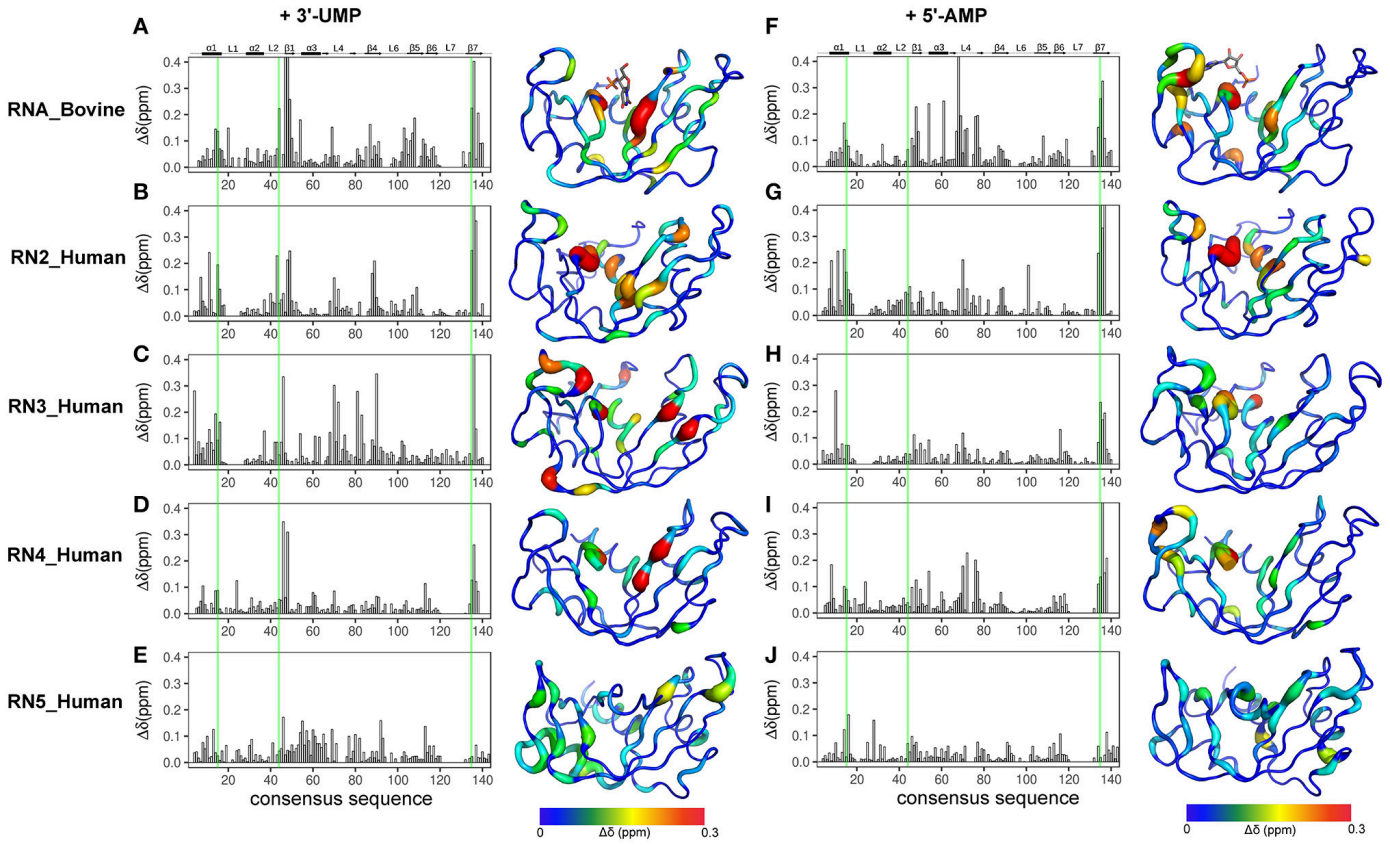
Effect of 3′-UMP and 5′-AMP ligand binding on functionally distinct RNases. Compounded chemical shift perturbations (Δδ_obs_ at the highest ligand concentration) relative to the apo form upon binding of two mononucleotides 3′-UMP **(A–E)** and 5′-AMP **(F–J)** for bovine RNase A **(A,F)** and human RNases 2 **(B,G)**, 3 **(C–H)**, 4 **(D,I)**, and 5 **(E,J)** are plotted as function of consensus sequence. Active-site residues (His12, Lys41, His119, RNase A numbering) are highlighted using green lines. Chemical shift perturbations were calculated by comparing the shifts at the largest enzyme:ligand molar ratios relative to the apo state for each enzyme. Δδ_obs_ are shown using the putty representation on the 3D structures to the right of the plots. Secondary structure of bovine RNase A is traced on top of the sequence alignment using blocks, arrows and dashed lines to represent α-helix, β-strand and loop regions, respectively. The 3′-UMP and 5′-AMP ligand positions, depicted on the structures of RNase A **(A,F)**, were obtained from PDB entries 1O0N and 1Z6S, respectively.

NMR chemical shifts are sensitive reporters of changes in the chemical environment of atoms and can be used to probe structural and conformational changes associated with ligand binding (Wishart et al., 1991; Case, 1998). This change in chemical shift can be detected as a shift in the peak of affected residues in the ^1^H-^15^N HSQC spectrum of a protein. Here, we performed NMR chemical shift titration experiments with increasing concentrations, up to saturation, of two ligands, 3′-UMP and 5′-AMP, to compare and characterize the effect of ligand binding on the conformational properties of the five RNases described above. The pyrimidine and purine mononucleotides are known to bind, respectively, to the B_1_ and B_2_ subsites in RNase A. Figure 3 shows the compounded chemical shift changes (Δδ_obs_ at the highest ligand concentration) upon binding of 3′-UMP (Figures 3A–E) and 5′-AMP (Figures 3F–J) for the five RNases as a function of the consensus sequence. The consensus sequence offers easier comparison of proteins with different sequence lengths by including gaps in the multiple sequence alignment. Chemical shift perturbations are also represented using a rainbow color scheme on the three-dimensional structures, shown to the right of the plots. Residues displaying large chemical shift perturbations (Δδ_obs_ > 0.1 ppm at the highest ligand concentration) are highlighted as spheres on the 3D structures of the different RNases in Figure S1. A list of residues displaying Δδ_obs_ > 0.1 ppm for all RNases is provided in Table S1.

Binding of 3′-UMP (Figures 3A–E) resulted in notable differences in the residues affected upon binding to the functionally distinct RNases. Large chemical shift perturbations were observed primarily near the B_1_ pyrimidine-binding subsite in most RNases, confirming the existence of an RNase A-like pyrimidine subsite in homologous RNases. RNase A (RNA_Bovine) displayed the largest 3′-UMP-induced chemical shift changes for residues near Thr45, His119 and Lys41, which directly interact with this ligand. Additional large perturbations (Δδ_obs_ > 0.1 ppm at the highest ligand concentration) were observed for some residues far from the active site (Figure S1), including residues of the β5 strand (consensus sequence positions 104–111) and Leu51 (α3), suggesting long-range conformational changes or motions in these regions upon ligand binding. The significant effect of 3′-UMP is consistent with the high binding affinity determined for RNase A (Table 1). RNase 2 (RN2_Human) showed fewer residues displaying large perturbations (15 residues with Δδ_obs_ > 0.1 ppm at the highest ligand concentration) relative to RNase A (21 residues with Δδ_obs_ > 0.1 ppm at the highest ligand concentration), consistent with the weaker binding affinity of the ligand for this enzyme relative to RNase A (Table 1). In addition to the chemical shift variations near the B_1_ subsite, RNase 2 also showed long-range effects of ligand binding for residues Ser64 and Lys66 in L4 and Cys37 of L2, suggesting potential conformational rearrangements triggered by 3′-UMP binding in these loop regions. RNase 3 (RN3_Human) showed perturbations dispersed throughout the protein with large chemical shift variations in loops L4 (positions 67–76) and L5 (positions 80–83), regions that were minimally perturbed in the other RNases upon 3′-UMP binding. The catalytic residues Lys38 and His128 showed no significant perturbations upon 3′-UMP binding, consistent with the lower dissociation constants determined here (Table 1) and reported previously for this enzyme (Gagné et al., 2012). The disperse but large (Δδ_max_ > 0.1 ppm at the highest ligand concentration) chemical shift perturbations, in addition to the observation that the displacement of some resonances upon 3′-UMP binding does not follow a straight line, suggests that this enzyme may either be experiencing conformational exchange in distal regions upon ligand binding and/or binding ligand in more than one site (Williamson, 2013). RNase 4 (RN4_Human), which experiences important and broadly distributed conformational exchange on the millisecond timescale in its apo form (Narayanan et al., 2017, 2018), showed few chemical shift perturbations upon ligand binding. As expected, these perturbations are primarily localized to the B_1_ pyrimidine binding site, correlating with a very low affinity for 3′-UMP (Table 1). The lack of significant perturbations is in agreement with the very low affinity of RNase 4 for 3′-UMP (*K*_*d*_ = 10,320 ± 622 μM). Interestingly, RNase 4 shows a much higher binding affinity for the oligonucleotide ligand dATATA (*K*_*d*_ = 72.9 ± 9 μM). We note that RNase 4 is the only member for which we observe such distinct binding affinities between single-nucleotide mimics of reaction products and a pentanucleotide DNA substrate analog. RNase 5 (RN5_Human) showed no effect in the B_1_ subsite while perturbations were observed far from the active site, suggesting weaker affinity and non-specific binding of the substrate, observations consistent with a previous report (Gagné et al., 2015b).

**Table 1:** Binding affinities of functionally distinct RNases for RNA and DNA ligands.

	***K_*d*_* 3′-UMP (μM)**	***K_*d*_* 5′-AMP (μM)**	***K_*d*_* dATATA (μM)^a^**
RNA_Bovine	9.7 ± 0.9^b^	124 ± 1^b^	N/A^c^
RN2_Human	455.9 ± 10.6	539.6 ± 31.4	383.9 ± 33.3
RN3_Human	460 ± 100	300 ± 45	302 ± 31
RN4_Human	10, 320±622	1, 324±72	72.9 ± 9
RN5_Human	1, 942±105	2, 476±77	N/A^c^

a *Single-stranded penta-deoxyribonucleotide (DNA) substrate analog of adenine and thymine nucleotide bases*.

b *Taken from Gagné et al. (2012)*.

c *Not available*.

Titration with 5′-AMP resulted in perturbations of residues near the purine binding site (B_2_) in all RNases, except RNase 5 (Figures 3F–J). Specifically, RNases A, 2 and 4 displayed significant perturbations in L4, residues of which were expected to interact with the purine base (Figure S1). RNase 2 showed additional perturbations in the N-terminal helix α1. RNase 3 showed fewer perturbed residues upon 5′-AMP binding relative to 3′-UMP binding, with residues localized primarily to the B_1_ subsite. The effects of ligand binding in loop 4 were diminished in RNase 3, with fewer residues and smaller magnitudes of perturbations observed upon ligand binding. RNase 4 showed large perturbations primarily in the L4 and the C-terminal regions. In contrast, RNase 5 showed the smallest perturbations among all RNases tested, with no changes in its significantly truncated loop 4, and very few residues displaying large chemical shift perturbations (Δδ_max_ > 0.1 ppm at the highest ligand concentration) throughout the protein upon ligand binding (Figure S1), suggesting few interactions with the ligand. These observations are in agreement with the low binding affinity determined for this enzyme (Table 1), and are consistent with previous observations (Gagné et al., 2015b). Overall, our results highlight the distinctive effects of ligand binding on the local chemical environment and/or potential changes in dynamic behavior for these different RNases. These observations illustrate that while all RNases share a similar structural fold, they display distinctly different conformational rearrangements upon binding of the two single-nucleotide RNA product mimics, further confirming their affinity differences and potential functional specialization.

### Comparative chemical shift analysis for RNases

**Figure 4:**
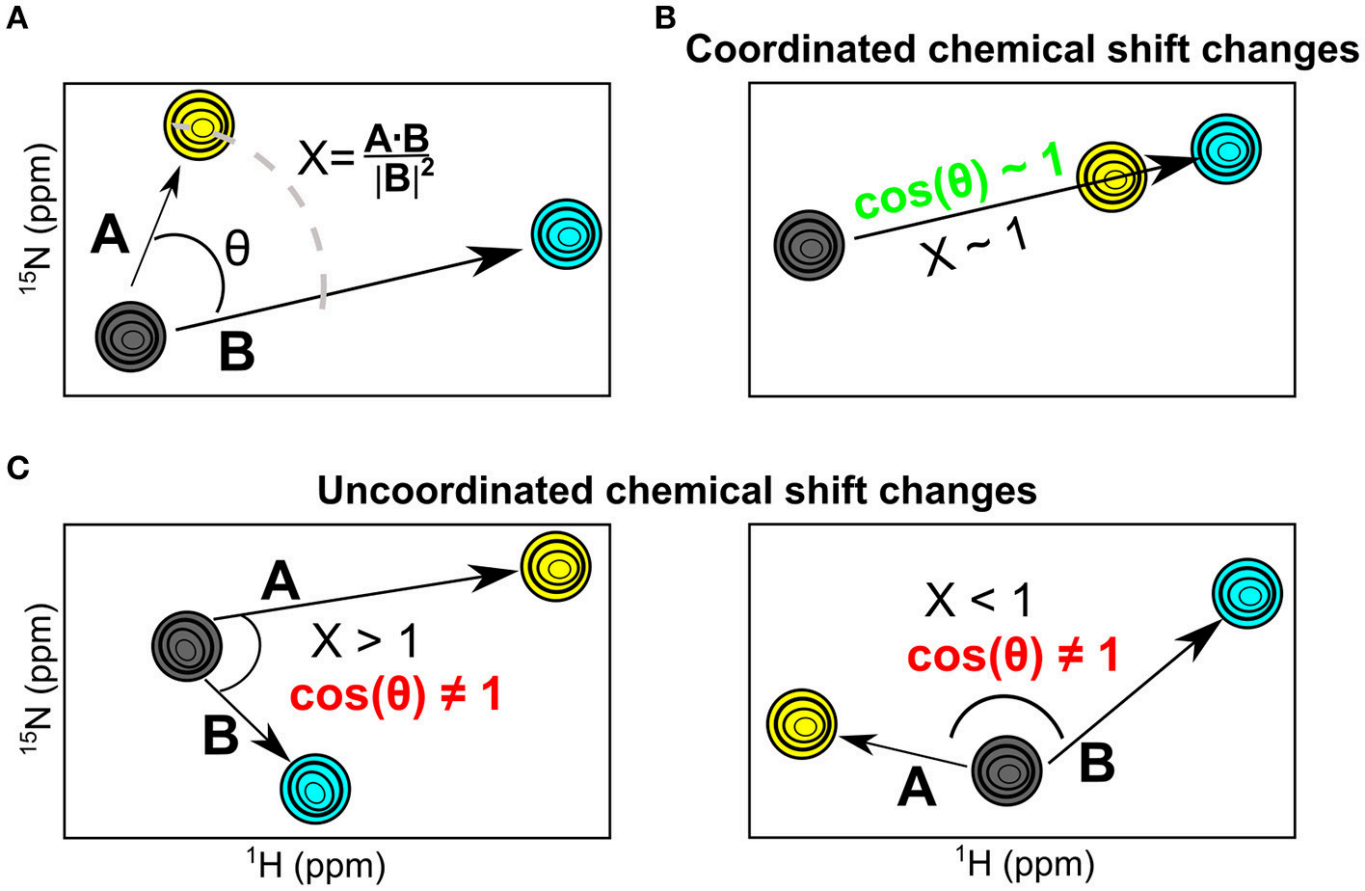
The NMR chemical shift projection analysis (CHESPA). **(A)** Schematic representation of the CHESPA analysis showing ^1^H-^15^N HSQC chemical shift resonances of the apo (gray), 3′-UMP (yellow) and 5′-AMP (cyan) states. Vectors A, B correspond to the compounded chemical shifts calculated for 3′-UMP-bound and 5′-AMP-bound complexes relative to the apo state. The two projection analysis parameters, projection angle and fractional shift, were calculated as described in the section Methods. **(B,C)** Graphical representation of scenarios representing coordinated **(B)** and uncoordinated displacements **(C)** of the ^1^H-^15^N HSQC chemical shifts upon ligand binding.

While chemical shift perturbations provide interesting information on local and long-range effects triggered by ligand binding to a protein, they nevertheless have limited comparative value when juxtaposing the effects of one system relative to another. In contrast, the chemical shift projection analysis (CHESPA) provides a systematic characterization of the effect of ligand binding or other perturbations on the magnitude and direction of the ^1^H-^15^N HSQC chemical shift displacements (Selvaratnam et al., 2012; Axe and Boehr, 2013; Gagné et al., 2015b). By characterizing the effect of two ligands that bind to distinct nucleotide subsites in the active site, we previously showed that this powerful technique can provide important insights into the long-range structural and dynamical changes associated with ligand binding (Gagné et al., 2015b). Here, we use the chemical shift projection analysis (CHESPA) to probe the coordinated changes associated with 3′-UMP and 5′-AMP binding to the five selected RNases. The schematic representation of the CHESPA approach is shown in Figure 4A. The projection angle (θ) is defined as the angle between the chemical shift peak displacements (for residues with Δδ_obs_ > 0.05 ppm at the highest ligand concentration) upon binding 3′-UMP (**A** vector) and 5′-AMP (**B** vector) relative to the apo state. The fractional shift (X) corresponds to the magnitude of displacement of **A** relative to **B**. Chemical shift changes are defined as coordinated when cos(θ) ≥ 0.9 and the chemical shift perturbations upon binding of the two ligands is similar, and uncoordinated when cos(θ) < 0.9 (Figures 4B,C).

**Figure 5:**
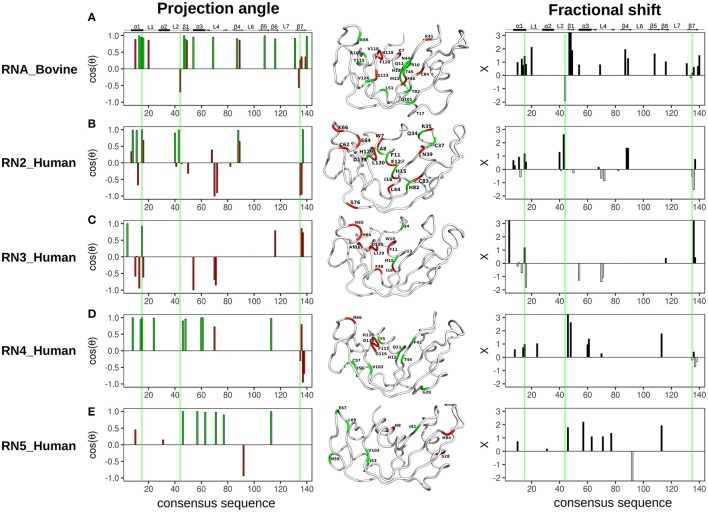
Chemical shift projection analysis of 3′-UMP and 5′-AMP ligand binding to functionally distinct RNases. The projection angle (cos(θ), left) and the fractional shift (X, right), calculated for residues with Δδ_obs_ > 0.05 ppm at the highest ligand concentration, are plotted as a function of consensus sequence for bovine RNase A and human RNases 2–5 upon binding of 3′-UMP and 5′-AMP single nucleotide RNA ligands. **(A–E)** Residues showing cos(θ) ~ 0.9 are colored green (coordinated), those with cos(θ) < 0.9 are colored red (uncoordinated). Residues are also identified on the three-dimensional structures of the five RNases using tube representations in the color scheme described above. Fractional shifts as a function of consensus sequence for bovine RNase A and human RNases 2–5, respectively, are shown on the right. Residues displaying positive and negative fractional shifts are displayed using black and white bars, respectively. Active-site residues (His12, Lys41, His119, RNase A numbering) are highlighted using green lines. Secondary structure of bovine RNase A is traced on top of the sequence alignment using blocks, arrows and dashed lines to represent α-helix, β-strand and loop regions, respectively.

The projection angle and fractional shift as a function of the consensus sequence determined for each of the five RNases is shown in Figure 5. For RNase A (RNA_Bovine), most residues involved in both 3′-UMP and 5′-AMP ligand binding, including Lys66 (P_0_), Thr45 (B_1_) and the catalytic His12, showed coordinated displacements (Figure 5A). Interestingly, active-site residues His119/Lys41, and Lys7, which form the P_1_ binding site, alongside Thr17 (loop 1) and residues of the C-terminal β7 strand showed uncoordinated displacements of the chemical shifts, indicating the different effects upon binding of the two ligands (Figure 5A). RNase 2 (RN2_Human) showed residues with both coordinated and uncoordinated displacements in the ligand binding sites, with residues of loop L4 displaying uncoordinated displacements (Figure 5B). In contrast to other RNases, RNase 3 (RN3_Human) showed predominantly uncoordinated displacements throughout the protein, with coordinated displacements observed only for Gln4 and the active site His15, suggesting a distinctly different effect of ligand binding in this enzyme (Figure 5C), consistent with the unusual behavior of 3′-UMP binding described above (Figure 3C). RNase 4 (RN4_Human) showed coordinated chemical shift displacements in the active site, while uncoordinated displacements were observed in the C-terminal β7 strand, similar to that of RNase A (Figure 5D). Interestingly, most residues displaying coordinated displacements are localized to the V2 domain of RNase 4. RNase 5 (RN5_Human) showed coordinated chemical shift displacements predominantly in the V_1_ domain, consistent with previous observations (Gagné et al., 2015b). Uncoordinated displacements were observed for residues His8, Ser28 and His84 (Figure 5E). Residues displaying coordinated chemical shift displacements also showed fractional shift ≥ 1, suggesting similar or larger changes in magnitude upon binding of 3′-UMP relative to 5′-AMP in all enzymes. Overall, our results suggest that while most of the amino acid residues that comprise the ligand binding pocket are identical in the primary and 3D structures of the selected RNases (Figure 2B), subtle variations in the active-site environment can lead to significantly different long-range effects throughout the structures upon ligand binding.

**Figure 6:**
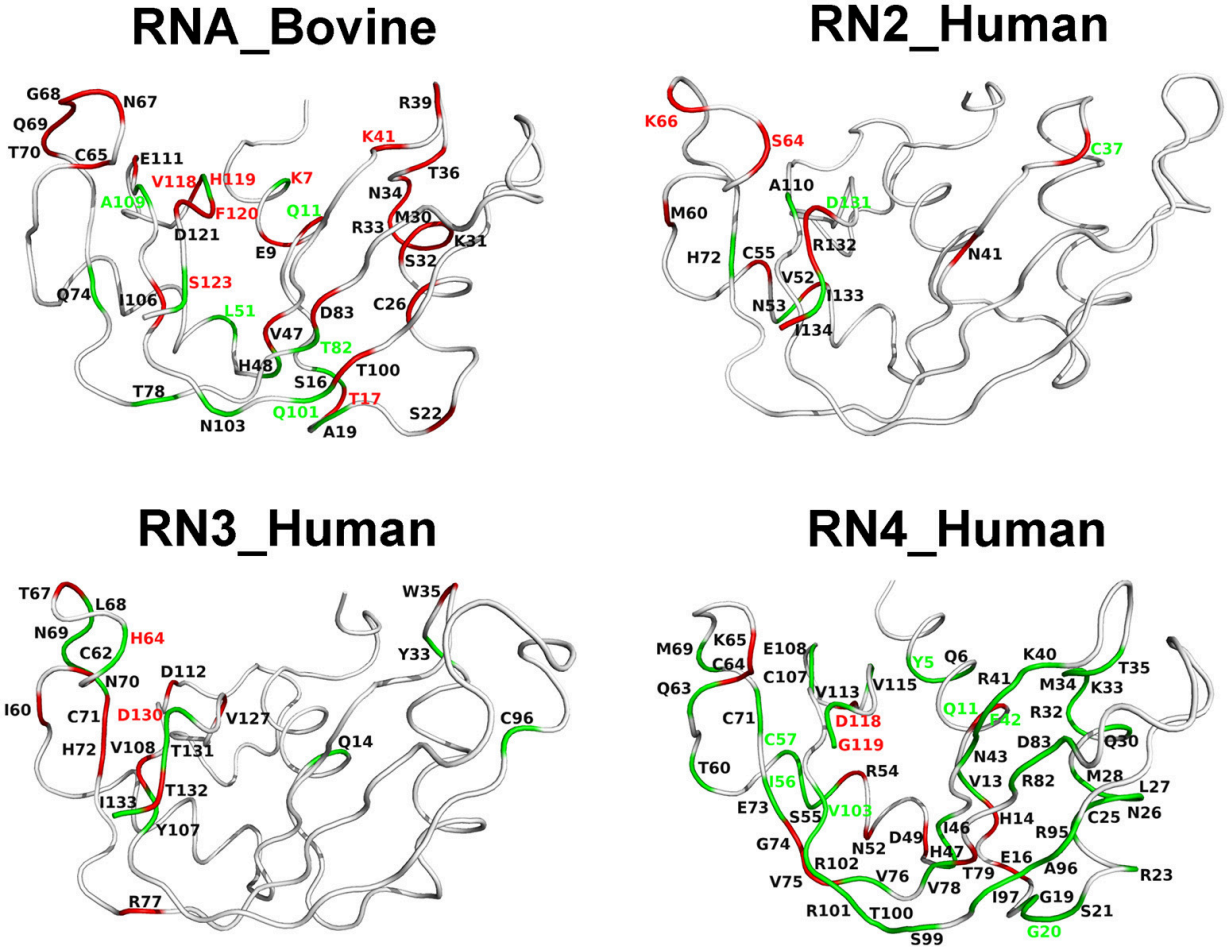
Coordinated conformational exchange and chemical shift perturbations in functionally distinct RNases upon ligand binding. Millisecond timescale dynamics was probed using ^15^N-CPMG relaxation dispersion experiments following binding of 3′-UMP and 5′-AMP to bovine RNase A (RNA_Bovine) and human RNases 2 (RN2_Human), 3 (RN3_Human), and 4 (RN4_Human). RNase 5 (RN5_Human) is not shown, as no measurable relaxation dispersion effects were observed upon ligand binding to this enzyme on this timescale. Residues displaying changes in conformational exchange upon binding (relative to the apo state) for *both* 3′-UMP and 5′-AMP are shown in green, while residues that show dynamical changes upon binding of *only one* of the two ligands are shown in red (see section Methods). Dynamical residues that also display coordinated or uncoordinated displacements in the chemical shift projection analysis (Figure 5) are highlighted using green and red residue labels, respectively.

Chemical shift perturbations report on changes upon ligand binding which may arise as a consequence of local/global structural rearrangements and/or changes in conformational motions in proteins. To gain insights into the potential role of dynamical changes in the observed chemical shift perturbations upon ligand binding, we used NMR ^15^N-CPMG relaxation dispersion experiments to identify residues that exhibit similar (or different) dynamical behavior upon binding of each of the two mononucleotide ligands (Figure 6). We note that RNase 5 (RN5_Human), which shows no measurable relaxation dispersion effects in the free and ligand-bound states, is not shown in Figure 6. Dynamical change is defined as the gain or loss of millisecond motions of any given residue, which displays a relaxation dispersion curve and Δ*R*_2_ > 2 s^−1^ (Figure S3), upon ligand binding relative to the apo state (see section Methods). Residues that display dynamical changes upon binding of *both* 3′-UMP and 5′-AMP ligands are shown in green, while residues which show dynamical changes upon binding of *only one* of the ligands are colored red. Further, residues displaying conformational exchange on the millisecond timescale that also show coordinated (uncoordinated) displacements in the chemical shift projection analysis (CHESPA) are identified using green (red) residue labels. Our results show that residues experiencing conformational exchange form significantly distinct dynamic clusters from one RNase member to the other, again supporting a link between millisecond timescale dynamics and divergent functional and biological specialization between these evolutionary distinct subfamily members. Also, while many residues display dynamical changes upon binding of the two ligands, only a subset of these residues exhibit significant chemical shift perturbations. Interestingly, most residues distal to the active site (defined as residues that are farther than 6 Å from the catalytic triad) that showed (un)coordinated CHESPA displacements (i.e., residues with red/green residue labels) also display (un)coordinated dynamical changes (red/green color of the cartoon loop) upon binding of the two ligands in the four RNases. For example, residues Thr17, Leu51, Thr82, and Gln101 in RNase A (RNA_Bovine); Lys66 of RNase 2 (RN2_Human); Tyr5, Gly20, Ile56, Cys57, and V103 of RNase4, which are distal from the active site, show (un)coordinated perturbations in chemical shifts and similar (un)coordinated dynamical changes upon binding the two ligands. This trend is not observed for some residues in the vicinity (< 6 Å) of the catalytic triad, such as Lys7, Gln11 in RNase A, Cys37, Asp131 in RNase 2; Asp130 in RNase 3; and Glu11, Asp118, and Gly119 in RNase 4. Exceptions to this trend include Ser123 of RNA_Bovine and His64 of RN3_Human, that display opposite trends to those described above for residues distal from the catalytic triad. We also note that the projection analysis is sensitive to changes in the ^1^H and ^15^N chemical shift changes while the dynamical changes are sensitive to the ^15^N chemical shift changes. Consequently, our analysis provides a qualitative comparison of the conformational exchange of residues between the apo and ligand-bound states. Nevertheless, these observations suggest that dynamical changes on functionally relevant timescales may be contributing to the coordinated displacements of distal residue networks in RNases. While further experiments are required to confirm this hypothesis, the qualitative comparison of the changes in the conformational exchange of residues presented in this study indicate that these events might correlate with long-range allosteric effects controlling the biological function of specific RNase subfamily members.

## Discussion

The role of conformational dynamics for optimal enzyme catalysis has emerged for a variety of enzyme systems (Narayanan et al., 2016). In their ligand-free states, several enzymes were shown to sample conformations observed upon ligand binding, suggesting that enzymes are primed for their catalytic function (Henzler-Wildman et al., 2007; Holliday et al., 2017; Kovermann et al., 2017). Ligand binding induces chemical shift changes in the protein, which may arise from changes associated with direct interaction with the ligand or conformational changes (dynamic and allosteric) induced by ligand binding (Williamson, 2013). While ligand-induced effects on structural and dynamical changes have been characterized for discrete enzymatic systems (Dhulesia et al., 2008; Gagné et al., 2015a; Doshi et al., 2016; Goricanec et al., 2016), these remain largely uncharacterized for most members within a superfamily. As a result, comparison of the effects of ligand binding between different members within a family that display distinct ligand specificities and catalytic efficiencies is lacking.

In this study, we combined chemical shift titration experiments with the chemical shift projection analysis and relaxation dispersion experiments to identify networks of amino acids displaying coordinated displacements upon binding of two mononucleotide ligands. The chosen ligands mimic cleavage products of a UpA dinucleotide RNA substrate and are known to bind to different nucleotide binding sites in bovine RNase A (Figure 1) (Nogués et al., 1995). Using select RNases corresponding to representative members of distinct phylogenetic subfamilies, we characterized the effect of binding and conformational exchange induced by these ligands to gain insights into the mechanism and functional biological differences observed in these structurally homologous RNases. Our results show that ligand binding induces different local and long-range effects on the various RNases, even for enzymes within the same subfamily, suggesting distinct conformational rearrangements upon ligand binding that may not be conserved in closely related RNases.

The binding affinity for a ligand, which relies on the subtle balance between substrate selectivity and avoiding being trapped with the bound ligand, among other factors, influences the catalytic efficiency of enzymes (Kovermann et al., 2017). Chemical shift perturbations dispersed far from the ligand binding site may be attributed to conformational rearrangements in the protein, assuming that a ligand binds to a single binding site (Williamson, 2013). Our results show that binding of the two mononucleotide ligands to the selected RNases triggers changes in distal regions in some of these enzymes. For example, binding of 3′-UMP induced effects in loops 4 and 5 and other regions distal from the active site in RNase 3, in addition to the non-linear chemical shift change upon addition of increasing concentrations of 3′-UMP, consistent with results from previous studies (Gagné et al., 2012). These observations suggest either conformational changes induced by ligand binding and/or binding to more than one site (Williamson, 2013). In contrast, RNase 4 showed very few perturbations, with residues displaying any significant changes localized to the pyrimidine binding site (Figure 3). These observations are consistent with the low affinity observed for this enzyme (*K*_*d*_ = 10,320 ± 622 μM for 3′-UMP). Interestingly, the low affinity for this 3′-UMP product analog does not correlate with the high binding affinity of a pentanucleotide single-stranded DNA substrate mimic (dATATA) to RNase 4 (Table 1). Considering the relatively low binding affinities of single-nucleotide product analogs, this observation suggests the existence of favorable synergistic subsite binding energies in the context of longer oligonucleotide substrates, and/or minimally that the 5-methyl moiety on the thymine pyrimidine DNA base provides favorable interactions with the enzyme that are absent in the homologous uracyl RNA nucleotide.

Amino acid residues belonging to the same allosteric network were previously shown to display coordinated chemical shift changes upon ligand binding or other perturbations (Selvaratnam et al., 2012). Using the CHESPA approach, we previously identified a network of amino acid residues involved in maintaining the structural stability of human angiogenin (RNase 5), highlighting long-range coordinated perturbations upon ligand binding. Here, we identified networks of amino acid residues that display coordinated chemical shift displacements upon binding of the two ligands in select RNases (Figure 5). Our results show that not all RNases display the same coordinated amino acid perturbations previously shown in RNase 5. Further, for RNases displaying coordinated displacements, the amino acid networks exhibiting such displacements are different. For example, while residue networks exhibiting coordinated displacements (defined in Figure 4B) in RNase A are localized primarily near the P_2_ and B_1_ binding sites (Figure 1), other RNases display significant differences in the localization of residues exhibiting coordinated changes upon binding of the two ligands. This lack of similarity in amino acid networks of coordinated displacements, even within closely related sequences such as RNases 2 and 3, suggests that the different RNases are uniquely adapted to their respective functions. We speculate that this may arise due to a variety of factors, including distinct nucleotide specificities. Further experiments probing the effect of binding a variety of nucleotides will provide insights into the contribution of nucleotide specificity to the observed effects.

In a recent study published from our group, we showed that the conservation of dynamical properties among RNase homologs correlates with their evolutionary conservation and shared biological functions (Narayanan et al., 2018). This previous work provided important insights into the role of conserved dynamical properties beyond enzyme catalysis. However, that study was performed on ligand-free states of enzymes and thus did not outline potential allosteric paths or conserved dynamic networks specific to subfamily members in the presence of ligands. Binding of a ligand can induce conformational rearrangements that may contribute to the observed chemical shift perturbations. In this work, we aimed to gain insights into the contribution of the conformational motions on the observed chemical shift perturbations by comparing CHESPA results with the coordinated changes in conformational exchange probed by ^15^N-CPMG relaxation dispersion experiments on the micro-millisecond timescale. The current study represents an additional step toward achieving the overarching goal of correlating the differences in dynamic/allosteric responses of distinct RNases with their diverse chemical and biological functions. Addressing this question on a broader functional and evolutionary scale requires the development of protein-specific allosteric modulators and/or mutagenesis studies that perturb the allosteric behavior of a targeted enzyme by directly affecting its ribonucleolytic activity or perturbing the other biological function in an independent fashion.

The present work illustrates a direct link between conformational perturbations and chemical shifts induced by ligand binding among structurally similar RNase homologs. Our results show that while not all residues displaying conformational exchange on the catalytically relevant millisecond timescale are reflected in the observed chemical shift perturbations, most residues displaying perturbations also exhibit coordinated dynamical changes (Figure 6). These observations highlight the contributions to observed chemical shift changes that arise from conformational rearrangements occurring upon ligand binding. Overall, these observations suggest that ligand-induced effects may be influenced, among other factors, by a combination of the different binding affinities and conformational dynamics of the different RNases. Further experiments are necessary to gain insights into the individual contribution of each factor to the observed effects on ligand binding in these functionally distinct RNases.

## Author contributions

ND, CN, and PKA conceived the research. CN performed all data analyses, with contributions from DNB and KB. DG performed experiments. CN and ND wrote the manuscript with contributions from DNB and PKA. All authors reviewed the manuscript.

### Conflict of interest statement

The authors declare that the research was conducted in the absence of any commercial or financial relationships that could be construed as a potential conflict of interest.
