# Supplementary material for: Ligand-Induced Variations in Structural and Dynamical Properties Within an Enzyme Superfamily
Source: Front Mol Biosci. 2018 Jun 12;5:54. doi: 10.3389/fmolb.2018.00054 (PMC6005897; doi:10.3389/fmolb.2018.00054)
Supplement: Supplementary file 1 [file Data_Sheet_1.pdf]

Supporting Information

Ligand-induced variations in structural and dynamical properties within an enzyme superfamily

Chitra Narayanan[1], David N. Bernard[1], Khushboo Bafna[2], Donald Gagné [1,†], Pratul K. Agarwal[3,4], Nicolas Doucet[1,5,*]

[1]INRS – Institut Armand-Frappier, Université du Québec, 531 Boulevard des Prairies, Laval, QC, H7V 1B7, Canada.

[2]Genome Science and Technology, University of Tennessee, Knoxville, TN 37996.

[3]Computational Biology Institute and Computer Science and Engineering Division, Oak Ridge National Laboratory, 1 Bethel Valley Road, Oak Ridge, TN 37830.

[4]Department of Biochemistry, Cellular and Molecular Biology, University of Tennessee, Knoxville, TN 37996.

[5]PROTEO, the Québec Network for Research on Protein Function, Engineering, and Applications, 1045 Avenue de la Médecine, Université Laval, Québec, QC, G1V 0A6, Canada.

[†]Present address: Structural Biology Initiative, CUNY Advanced Science Research Center, New York, NY, 10032.

*To whom correspondence should be addressed: Nicolas Doucet (nicolas.doucet@iaf.inrs.ca)

Table S1. List of residues displaying $\Delta\delta_{obs} \geq 0.1$ ppm upon 3'-UMP and 5'-AMP binding to the five RNases

Residues displaying Δδ>=0.1 ppm upon UMP binding									
RNA_Bovine		RN2_Human		RN3_Human		RN4_Human		RN5_Human	
Residue	Δδ, ppm	Residue	Δδ, ppm	Residue	Δδ, ppm	Residue	Δδ, ppm	Residue	Δδ, ppm
11	0.145	7	0.147	4	0.281	5	0.105	11	0.125
12	0.137	11	0.241	11	0.134	20	0.125	42	0.172
17	0.149	15	0.195	14	0.194	42	0.349	50	0.113
41	0.223	16	0.102	16	0.162	44	0.310	51	0.157
44	0.490	37	0.229	31	0.129	103	0.114	53	0.120
45	1.197	42	0.213	40	0.335	116	0.127	56	0.124
46	0.258	43	0.247	48	0.118	117	0.261	61	0.107
47	0.110	64	0.145	55	0.109	118	0.121	65	0.125
51	0.180	66	0.101	57	0.105			72	0.106
66	0.152	82	0.162	62	0.123			84	0.159
82	0.163	83	0.209	64	0.302			103	0.137
96	0.149	103	0.108	66	0.238				
97	0.108	129	0.249	69	0.113				
99	0.101	130	0.477	75	0.279				
100	0.130	131	0.361	77	0.189				
101	0.187			84	0.346				
105	0.107			129	0.568				
119	0.225			130	0.136				
120	0.403								
122	0.206								

Residues displaying Δδ>=0.1 ppm upon AMP binding									
RNA_Bovine		RN2_Human		RN3_Human		RN4_Human		RN5_Human	
Residue	Δδ, ppm	Residue	Δδ, ppm	Residue	Δδ, ppm	Residue	Δδ, ppm	Residue	Δδ, ppm
11	0.166	7	0.101	10	0.279	5	0.183	12	0.122
43	0.107	8	0.208	41	0.112	11	0.100	14	0.179
45	0.227	11	0.244	64	0.118	20	0.118	25	0.158
46	0.133	14	0.250	110	0.132	44	0.125	106	0.106
51	0.239	15	0.164	128	0.235	50	0.158	117	0.112
58	0.249	39	0.109	129	0.169	66	0.178		
64	0.134	64	0.211	130	0.194	68	0.228		
65	0.450	66	0.101			70	0.201		
66	0.193	82	0.100			71	0.157		
68	0.139	83	0.105			115	0.110		
69	0.145	95	0.190			116	0.137		
71	0.190	128	0.236			117	0.625		
72	0.194	129	0.460			118	0.152		
101	0.116	130	0.332			119	0.208		
112	0.104	131	0.464						
118	0.149								
119	0.259								
120	0.325								
121	0.107								

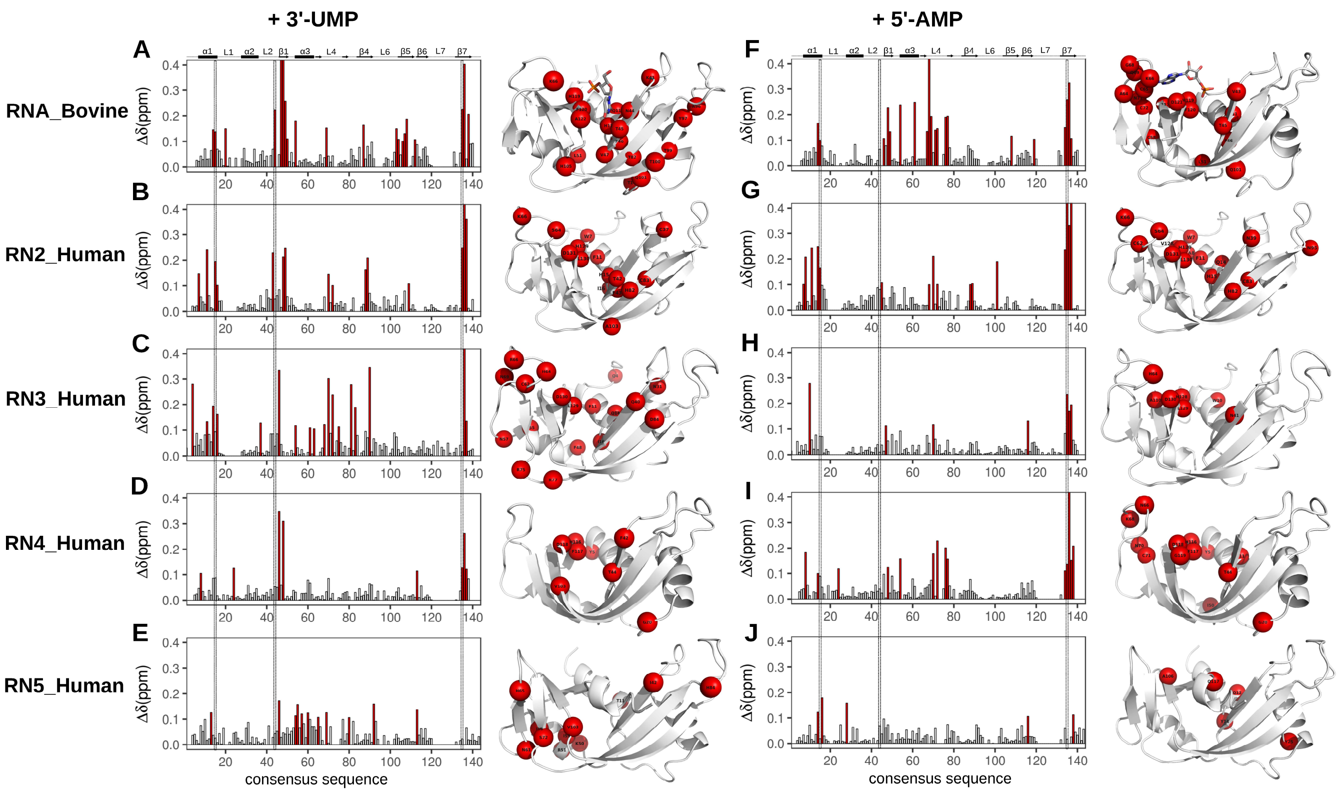

Figure S1. Chemical shift perturbations upon ligand binding to RNases. Compounded chemical shift variations ($\Delta\delta_{obs}$ at the highest ligand concentration) relative to the apo form upon binding of two mononucleotides 3'-UMP (A-E) and 5'-AMP (F-J) for bovine RNase A (A,F) and human RNases 2 (B,G), 3 (C-H), 4 (D,I) and 5 (E,J) are plotted as function of consensus sequence. Residues displaying $\Delta\delta_{obs}$ greater (lower) than 0.1 ppm are shown as red (white) bars. Active-site residues (His12, Lys41, His119, RNase A numbering) are highlighted using dashed lines. Residues with $\Delta\delta_{obs}$ > 0.1 ppm at the highest ligand concentration are depicted as spheres on the three-dimensional structures to the right of the plots.

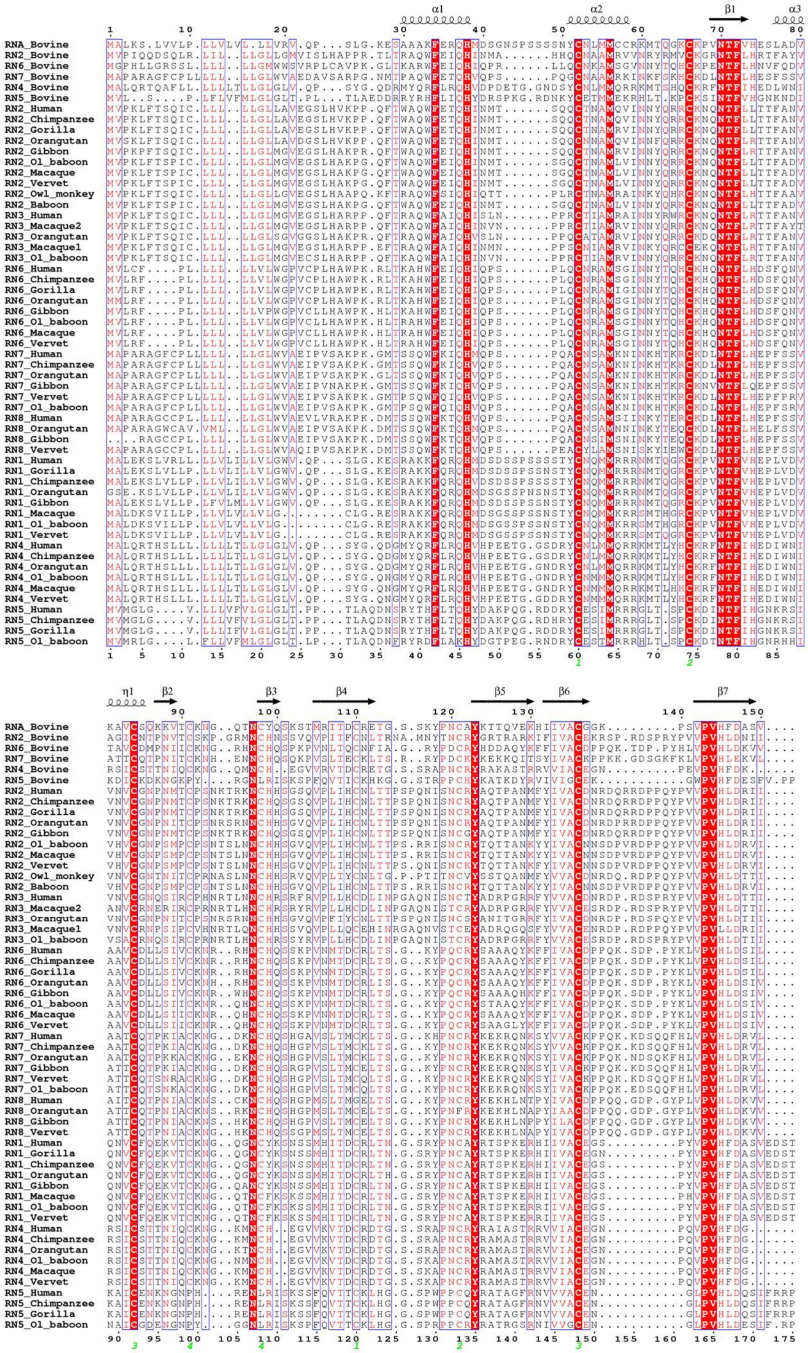

Figure S2. Multiple sequence alignment of bovine and Hominidae RNases 1-8. The primary structure numbering for bovine RNase A (RNA_Bovine) and the consensus sequence numbering are shown above and below the alignment, respectively. The α-helical and β-strands are identified above the alignment. Cysteine disulphide linkages are identified in green below the consensus sequence numbering.

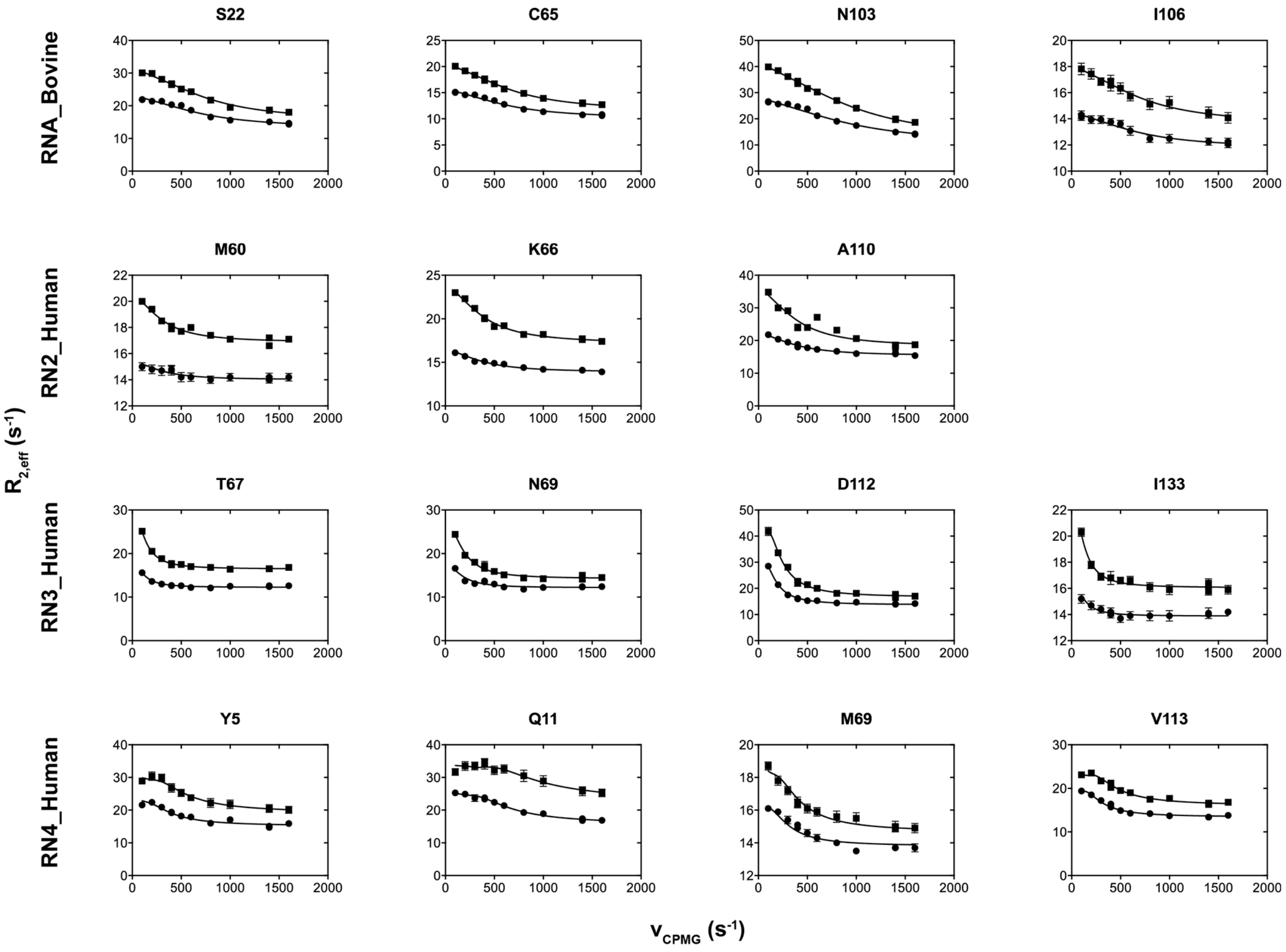

Figure S3. Representative ^{15}N-CPMG relaxation dispersion curves for the different RNases. Relaxation dispersion curves of select residues from the four RNases that display conformational exchange in the apo state. As RN5_Human does not display any such behavior, no curves are shown for this enzyme. Squares and circles respectively correspond to data recorded at 800 MHz and at 600 MHz (RNA_Bovine) or 500 MHz (RN2, RN3 and RN4_Human). Data were dual-fitted to the full Carver-Richards relaxation dispersion equation. Residues displaying (un)correlated dynamical changes were determined as described in the Methods section of the manuscript.
